# Rutaecarpine Attenuates Oxidative Stress-Induced Traumatic Brain Injury and Reduces Secondary Injury *via* the PGK1/KEAP1/NRF2 Signaling Pathway

**DOI:** 10.3389/fphar.2022.807125

**Published:** 2022-04-12

**Authors:** Min Xu, Liu Li, Hua Liu, Wei Lu, Xiaoyang Ling, Mingjie Gong

**Affiliations:** ^1^ Department of Neurosurgery, Kunshan Hospital of Traditional Chinese Medicine, Kunshan Affiliated Hospital of Nanjing University of Chinese Medicine, Kunshan, China; ^2^ Collaborative Innovation Center of Jiangsu Province of Cancer Prevention and Treatment of Chinese Medicine, Nanjing University of Chinese Medicine, Nanjing, China; ^3^ Department of Neurosurgery, Affiliated Kunshan Hospital of Jiangsu University, Kunshan, China; ^4^ Department of Neurosurgery, Changshu No.2 People’s Hospital, The Affiliated Changshu Hospital of Xuzhou Medical University, Changshu, China

**Keywords:** traumatic brain injury, rutaecarpine, PGK1, Nfr2, oxidative stress

## Abstract

The oxidative stress response caused by traumatic brain injury (TBI) leads to secondary damage in the form of tissue damage and cell death. Nuclear transcription-related factor 2 (NRF2) is a key factor in the body against oxidative stress and has an important role in combating oxidative damage in TBI neurons. In the present study, we investigated whether rutaecarpine could activate the PGK1/KEAP1/NRF2 pathway to antagonize oxidative damage in TBI neurons. We performed controlled cortical impact (CCI) surgery on mice and taken H_2_O_2_ treatment on PC12 cells to construct TBI models. The results of western blot showed that the expression of PGK1, KEAP and NRF2 was regulated and accompanied by altered levels of oxidative stress, and the use of rutaecarpine in the TBI model mice significantly improved cognitive dysfunction, increased antioxidant capacity and reduced apoptosis in brain tissue. Similar antioxidant damage results were obtained using rutaecarpine in a PC12 cell model. Furthermore, through the use of the protein synthesis inhibitor CHX and the proteasome synthesis inhibitor MG-132, rutaecarpine was found to promote the expreesions of PGK1 and NRF2 by accelerating PGK1 ubiquitination to reduce PGK1 expression. Therefore, rutaecarpine may be a promising therapeutic agent for the treatment of TBI-related neuro-oxidative damage.

## Introduction

Traumatic brain injury (TBI) has a high rate of death and disability and is a global public health problem (Marmarou, 2003). Cranial trauma can lead to direct mechanical damage to neurons, glial cells and blood vessels (Mustafa and Alshboul, 2013; McGinn and Povlishock, 2016). Subsequently, a secondary injury cascade response is triggered, ultimately leading to cell death and tissue damage, and is the most important cause of neurodegeneration and neurological dysfunction after TBI (Pearn et al., 2017). Studies have demonstrated that patients with TBI develop early cerebral ischaemia, and that ischemic injury induces the production of reactive oxygen species (ROS), which subsequently leads to mitochondrial dysfunction and DNA cleavage, triggering neuronal apoptosis (Brown et al., 2006; Launey et al., 2020). Although research into the pathophysiology of TBI have progressed rapidly, the drugs currently used for neurocytoprotection have not led to satisfactory therapeutic outcomes and the molecular mechanisms behind them are not fully understood (Skolnick et al., 2014; Zhang et al., 2017).

Nuclear transcription-related factor 2 (NF-E2 related factor 2, NFR2) is a key factor in the body’s resistance to oxidative stress (Shah et al., 2018). Under normal conditions, NFR2 is localized in the cytoplasm and binds via the Nrf2-ECH homology (Neh2 structural domain) to the double glycine repeat (DGR) region of Kelch-1ike ECH- associated protein l (Keap1), which can negatively regulate NRF2 expression through cullin-3 (CUL3)-mediated ubiquitination and proteasomal degradation (Namani et al., 2014; Namani et al., 2018). In response to oxidative stress, Keap1-Nrf2 binding is destabilised and Nrf2 is released and transferred to the nucleus where it binds to the antioxidant response element (ARE), activating the transcription of downstream genes and translating antioxidant proteins, thereby protecting the body from oxidative stress (Liu et al., 2017; Hu et al., 2018).

Rutaecarpine is derived from the *Euodia rutaecarpa (Juss.) Benth.*, and it has good permeability to the blood-brain barrier (Zhang et al., 2018). Recent studies have shown that rutaecarpine can target and activate the NFR2/HO-1 pathway to reduce craniofacial injury (Yan et al., 2013; Han et al., 2019). In addition, rutaecarpine can attenuate cerebral ischemia-reperfusion-induced neuronal injury by inhibiting neuronal apoptosis (Han et al., 2019). The aim of this study is to determine whether rutaecarpine can activate the NFR2 signaling pathway to antagonize the oxidative damage to neurons caused by TBI.

## Materials and Methods

### Reagents and Antibodies

The antibodies to cleaved caspase3 (no. 9661), caspase3 (no. 9662), Bcl2 (no. 2764), Bax (no. 14796s), NRF2 (no. 12721), PGK1 (no. 68540), HO-1 (no. 86806), Lamin B (no. 13435), GAPDH (no. 5174) and Anti-rabbit IgG, HRP-linked Antibody (no. 7074) were purchased from Cell Signaling Technology. Cycloheximide (CHX, no. 2112) was obtained from Cell Signaling Technology. Catalase (CAT) assay kit (no. A007-1-1) and Superoxide Dismutase (SOD) assay kit (no. A001-3-2) were obtained from Nanjing Jiancheng Bioengineering Institute. DCFH-DA (no. S0033M) purchased from Beyotime Biotechnology was used to detected the level of ROS in PC12 cells. Rutaecarpine (purity ≥ 98%, R817266) purchased from Macklin Inc.

### Animals and Experimental Protocols

Specific pathogen free (SPF) male C57BL/6 mice (weighing 20–25 g) were purchased from Jiangsu ALF Biotechnology Co. In this study, we were used a controlled cortical impact (CCI) induced-TBI mouse model. The mice were housed in a warm room with a room temperature of 20–25°C and 40%–60% humidity, with 12 h of daily light and free access to food and water. All animal procedures were carried out in accordance with the Medical Ethics Committee of Nanjing University of Chinese Medicine (No. 202102A002). After 1 week of acclimatization, mice were randomly divided into control group (sham), model group (TBI), 5-Rut group (5 mg/kg rutaecarpine), 10-Rut group (10 mg/kg rutaecarpine) and 20-Rut group (20 mg/kg rutaecarpine), with 10 mice in each group (Han et al., 2019).

The TBI mouse model was prepared according to Feeney’s free fall method reported in a previous study (Wu et al., 2014). Briefly, mice were anaesthetized by intraperitoneal injection of sodium pentobarbital, fixed in a brain stereotaxic apparatus and the site of injury was identified: 2 mm to the left of the midline of the skull and 2 mm anterior to the herringbone suture. A circular hole approximately 2 mm in diameter was drilled through in the skull of mice and weight was thrown down in a free fall motion on a vertical tube 20 cm from the hole (impact force 500 g/cm) to impact the area of the circular hole. The mice in the control group were only drilled through the skull, without the impact of the weight.

Mice in the rutaecarpine-treated groups were injected intraperitoneally with rutaecarpine (5, 10, 20 mg/kg) 24 h and 30 min before surgery, 2, 24, 48 and 72 h after surgery, respectively, while mice in the control and model groups were given equal volumes of saline. In addition, mice were divided into CHX group (mice received Feeney’s free fall and 20 mg/kg Cycloheximide injection) and CHX+ rutaecarpine group (mice received Feeney’s free fall and 20 mg/kg Cycloheximide and 20 mg/kg rutaecarpine injection). Mice were euthanized by injection of 200 mg/kg pentobarbital sodium 2 h after the last dose and brain tissues were removed for testing of relevant indicators.

### Neurological Function Tests

The modified Neurological Severity Scores (mNSS) was used to assess the degree of neurological impairment in mice (Chen et al., 2001), consisting of four components: motor, sensory, balance and reflex tests. The total score of the mNSS test is 18, with a score of 0 indicating normal; 1 to 6 indicating mild impairment; 7 to 12 indicating average moderate impairment; and 13–18 indicating severe injury.

### Evans Blue Tests the Permeability of the Blood-Brain Barrier

Evans Blue (EB) has a high binding rate to plasma albumin in blood, but plasma proteins cannot cross the blood-brain barrier (BBB), and when the BBB is disrupted, EB can enter and color the nervous system. Prior to sacrifice, mice were injected with 2% EB in the tail vein, the mice were anaesthetized, then the thorax opened and intracardially instilled with heparin saline. Injured lateral brain tissue was weighed and incubated with dimethylamide at 60°C for 24 h, and the absorbance at 620 nm was measured by spectrophotometer. The amount of EB in the brain was calculated from the standard curve of EB.

### Activities Assay for CAT and SOD

The tissue was weighed and added to saline at a ratio of weight (g): volume (ml) = 1:9 and a 10% tissue homogenate was made with ice bath. The reaction of CAT decomposing H_2_O_2_ can be rapidly terminated by the addition of ammonium molybdate, with the remaining H_2_O_2_ producing a yellow complex with ammonium molybdate. CAT viability was calculated by CAT kit according to the manufacturer’s instructions, mixing the reaction at 37°C for 1 min and measuring the value of OD_405_. SOD viability was calculated from the OD value measured at 450 nm after incubation at 37°C for 20 min with the SOD kit reagent mixed.

### Hematoxylin-Eosin Staining

Briefly, the brain tissues were fixed in 4% paraformaldehyde for 24 h and then embedded in paraffin. The embedded tissues were cut into 4-μm thick sections, dewaxed and dehydrated and stained with hematoxylin and eosin. Sections were observed under a light microscope (Olympus, Japan).

### Immunofluorescence Assay

Paraffin sections were prepared from injured lateral brain tissue, dewaxed twice in xylene and subsequently hydrated in 100%, 95%, 85% and 75% alcohol solutions; antigen repair was followed by permeabilization with 1% Triton X-100 for 30 min. Sections were incubated with Anti-Cleaved Caspase-3 (Asp175) Rabbit mAb (9664, CST) and Anti-NeuN (ab177487, Abcam) antibody at 4°C overnight, and DAPI was added to stain nuclei for 15 min, anti-fluorescence quencher for blocking, placed under a fluorescent microscope and photographed.

### Western Blot Assay

Western blotting was performed according to standard methods. In brief, cells or tissues wee lysed in a buffer containing protease inhibitors. After protein concentration has been determined with a BCA kit (P0012S, Beyotime), equal amounts of proteins were separated in 10% SDS-PAGE gels and then transferred to PVDF membranes. Membranes were incubated overnight at 4°C with primary antibodies and for 2 h at 37°C temperature with secondary antibody, the bands were analyzed for greyness using ImageJ software after development with extremely hypersensitive ECL chemiluminescent reagent (P0018FS, Beyotime). In addition, nuclear extracts were prepared by a Nuclear Protein Extraction Kit (R0050, solarbio) for experiments involving nuclear proteins. Lamin B was used as an internal control for Nucleus-NRF2, and GAPDH was used as an internal control for remaining proteins.

### Cell Culture and Grouping

PC12 cells were obtained from Nanjing University of Chinese Medicine. These cells were cultured in Dulbecco’s modified Eagle medium (DMEM) containing 10% fetal bovine serum (FBS) and 100 U/ml penicillin and 0.1 mg/ml streptomycin (C0222, Beyotime). First, we determined the modeling concentration of H_2_O_2_ was 300 μM and the administration concentrations of rutaecarpine were 0.2, 0.4 and 0.8 μM, respectively, based on the survival rate at different concentrations of H_2_O_2_ and rutaecarpine as shown in Figures 4A,B.

Cells were divided into 5 groups: control group, model group, 0.2-Rut group, 0.4-Rut group and 0.8-Rut group. In the control group, only an equal volume of culture medium at was used. The model group received only oxidative stress treatment with 300 μM of H_2_O_2_, but the 0.2-Rut group, 0.4-Rut group and 0.8-Rut group were treated with 300 μM of H_2_O_2_ while incubated with 0.2, 0.4 and 0.8 μM rutaecarpine, respectively.

### Cell Viability Assay

PC12 cells in logarithmic growth phase were spread in 96-well plates at 5 × 10^3^ cells per well. After 24 h of adherent culture, the cells were further incubated with 0.2, 0.4 and 0.8 μM rutaecarpine for 48 h. Cell viability was determined by MTT assay (M1020, Solarbio, China) according to the manufacturer’s instructions.

### Detection of ROS

The ROS content of the PC12 cells was tested by H2DCF-DA probe (S0033M, Beyotime) according to the manufacturer’s instructions. ROS within the cells can oxidize non-fluorescent DCFH to generate fluorescent DCF. The treated cells were incubated with 10 μM DCF-DA probe for 20 min at 37°C and then washed with serum-free cell culture medium. The fluorescence spectrum of DCF is similar to that of FITC. Using the parameter settings for DCF detection by FITC, and the fluorescent intensity of DCF in the cells can be detected in real time with an excitation wavelength of 488 nm and an emission wavelength of 525 nm.

### The Detection of Apoptosis Ratio

Cells were first digested with 0.25% trypsin and collected, and cell density was controlled at 5 × 10^5^ cells after washing the cells with PBS. Cells were then mixed with 500 μL of Binding Buffer, 5 μL of Annexin V-FITC was added, and followed by 5 μL of Propidium Iodide for 15 min. The apoptosis was detected by flow cytometry with green fluorescence of Annexin V-FITC and red fluorescence of PI.

### Statistical Analysis

Statistical analyses were performed using Graph Pad 8.02 software. Data were expressed as the means ± standard deviation (SD) of three independent experiments. Student’s t-test was performed to evaluate differences between two groups, and one-way analysis of variance (ANOVA) with Tukey’s test was used for comparison among multiple group. *p* < 0.05 was considered significant.

## Results

### Rutaecarpine Attenuates Oxidative Stress Damage in TBI Neurons of TBI Mice Models

**FIGURE 1:**
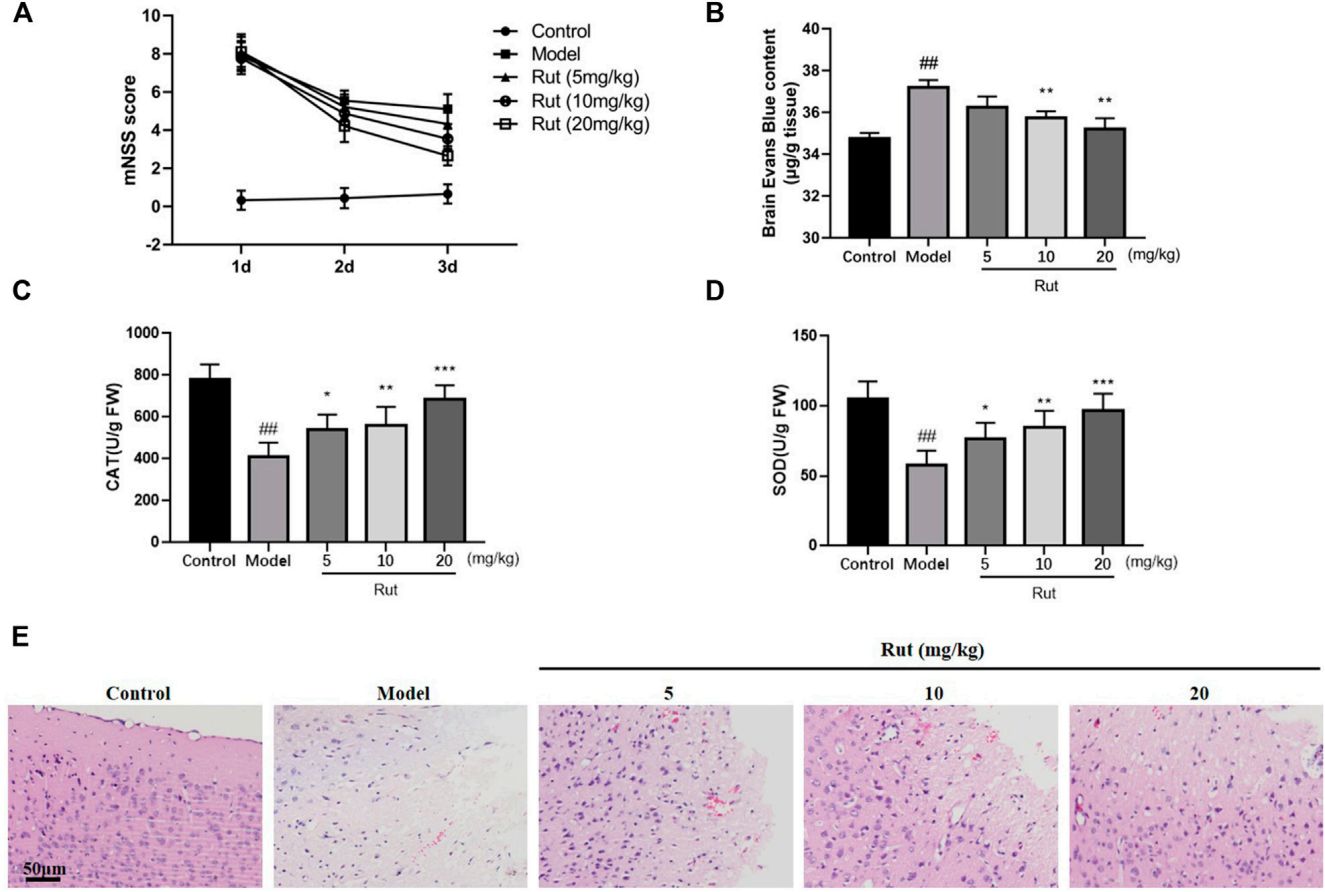
Rutaecarpine attenuates oxidative stress damage in TBI neurons of TBI mice models. **(A)** Neurological function scores in mice. **(B)** Blood-brain barrier permeability in mice brain tissues. The activity of **(C)** CAT and **(D)** SOD of mice in each group. **(E)** HE staining of damaged areas of mouse cerebral cortex. Compared with the control group, #*p* < 0.05 and ##*p* < 0.01; compared with the model group, *p < 0.05 and **p < 0.01.

We first detected whether rutaecarpine has protective effect on controlled cortical impact (CCI)-induced TBI mice, the mNSS tests were performed and results were showed in Figure 1A. The mNSS scores of the TBI mice were significantly higher than those of the Control group. At 24 h post-TBI, there was no significant difference between the mNSS scores of the TBI model group and the rutaecarpine administration group. The mNSS scores of all TBI groups decreased over time, with more pronounced downward trends in the 5, 10, and 20 mg/kg Rutaecarpine treatment groups compared to the Model group at 72 h post-TBI. As shown in Figure 1B, Evans Blue test was performed and TBI mice treated with rutaecarpine had lower Evans Blue exudation than mice in the TBI model group after CCI surgery, indicating that rutaecarpine improved the extent of TBI-induced neurological damage and BBB disruption. Furthermore, we determined whether rutaecarpine reduced TBI-induced oxidative stress by assaying CAT and SOD. The results showed that CAT and SOD also increased significantly with increasing concentration of rutaecarpine (*p* < 0.05, Figures 1C,D). In Figure 1E, HE staining results showed that TBI resulted in significant brain oedema and cell rupture, while the rutaecarpine administration group was less impaired.

### Rutaecarpine Inhibits Apoptosis in Neuronal Cells of TBI Mice Models

**FIGURE 2:**
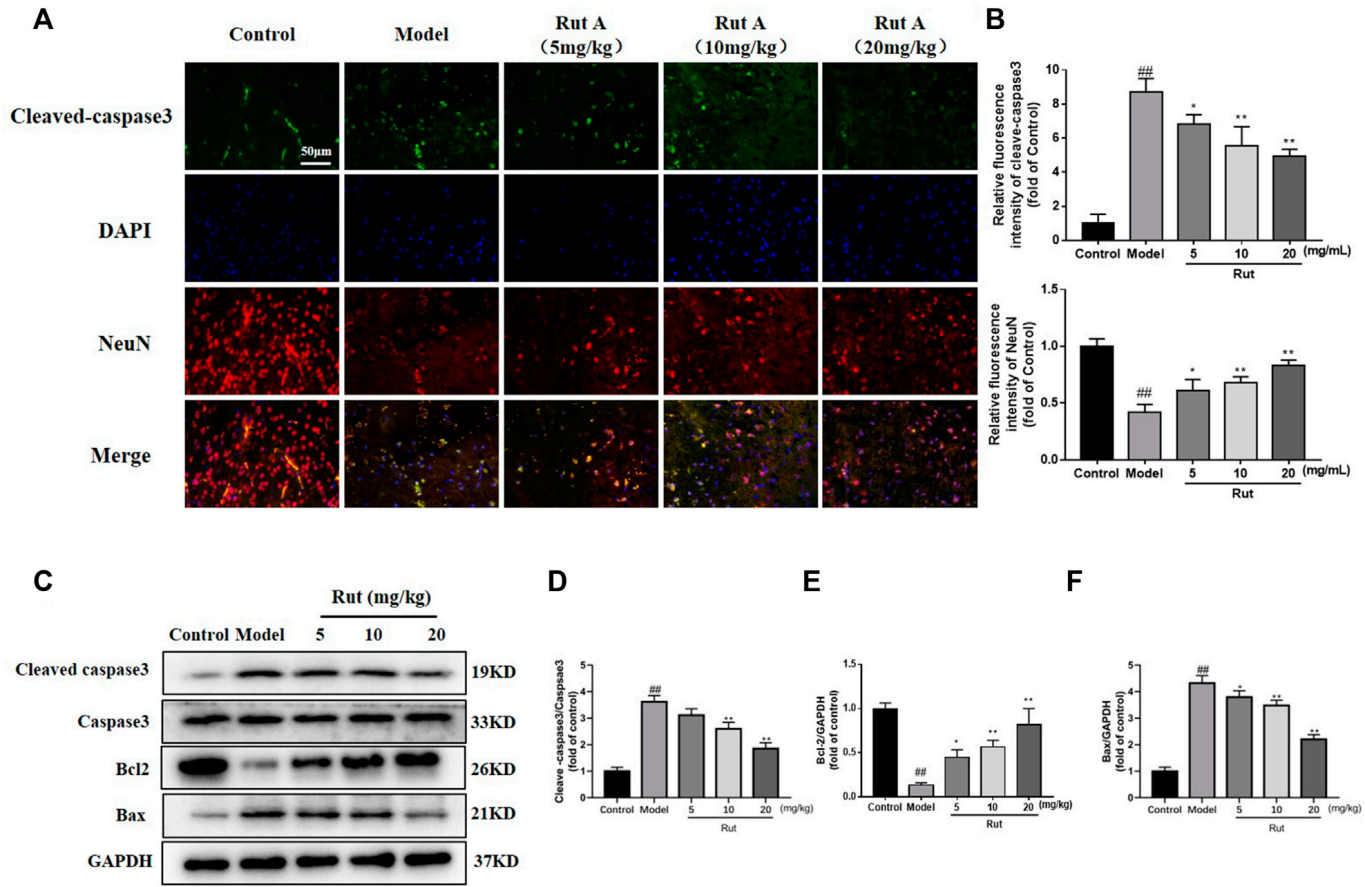
Rutaecarpine inhibits apoptosis in neuronal cells of TBI mice models. **(A)** Detection of neuronal apoptosis in mouse cerebral cortex by cleaved-Caspase-3 and DAPI immunofluorescence staining. **(B)** Relative intensity of immunofluorescence of cleaved-Caspase-3 and NeuN. **(C)** Expressions of apoptosis-related proteins cleaved-Caspase-3, Caspase-3, Bcl-2 and Bax in cerebral cortex. Relative expressions of **(D)** cleaved-Caspase-3/Caspase-3, **(E)** Bcl-2 and **(F)** Bax in each group. GAPDH was used as the internal control for proteins in **(C)**. Compared with the control group, #*p* < 0.05 and ##*p* < 0.01; compared with the model group, *p < 0.05 and **p < 0.01.

Apoptosis was associated with TBI, and we examined apoptosis in brain tissue by immunofluorescence and Western Blot. As shown in Figures 2A,B, the expression of cleaved-caspase3 was significantly increased in the brain tissue of TBI mice compared to controls, while rutaecarpine significantly decreased the expression of cleaved-caspase3. More results on protein expression were shown in Figures 2C–F, the expressions of cleaved-caspase3/caspase3 and Bax were increased and the expression of Bcl-2 was decreased in the model group, while rutaecarpine reversed this change.

### Rutaecarpine Regulates Oxidative Damage in TBI Mice Neurons Through Activation of the PGK1-NRF2 Signaling Pathway

**FIGURE 3:**
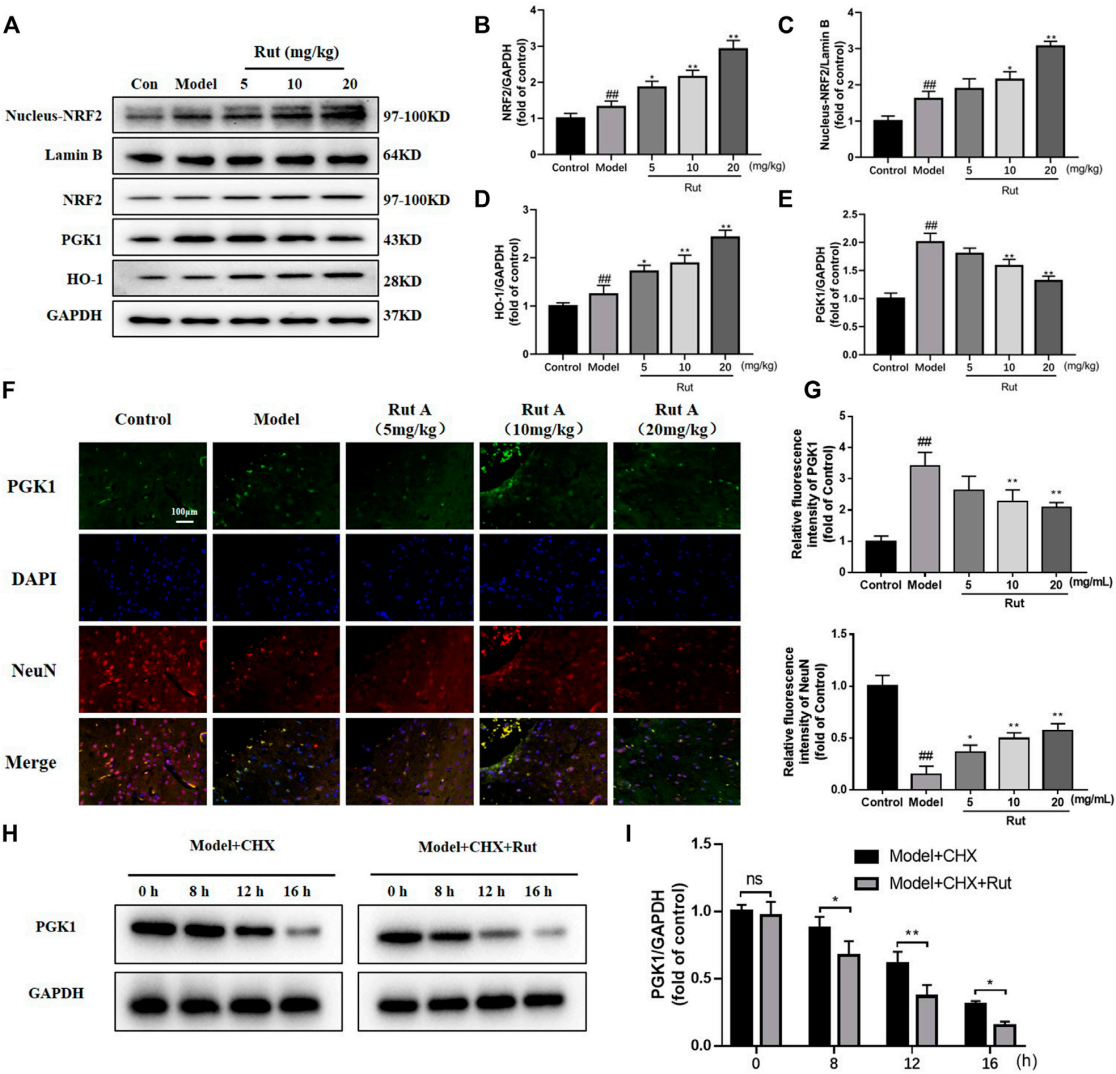
Rutaecarpine regulates oxidative damage in TBI mice neurons through activation of the PGK1-NRF2 signaling pathway. **(A)** Expressions of PGK1-NRF2 pathway-related proteins NRF2, Nucleus-NRF2, HO-1 and PGK-1 in cerebral cortex. Relative expressions of **(B)** NRF2, **(C)** Nucleus-NRF2, **(D)** HO-1 and **(E)** PGK-1. **(F)** Representative images of PGK1 and DAPI immunofluorescence staining. **(G)** Relative intensity of immunofluorescence of PGK1 and NeuN. **(H)** With the treatment of CHX, rutaecarpine further reduced the expression of PGK1. **(I)** The relative expression of PGK1 was presented in histograms. Lamin B was used as an internal control for Nucleus-NRF2, and GAPDH was used as an internal control for remaining proteins in **(A)**. Compared with the control group, #*p* < 0.05 and ##*p* < 0.01; compared with the model group, *p < 0.05 and **p < 0.01.

It has been demonstrated that NFR2 expression decreases progressively along with neuronal development, making neurons more sensitive to oxidative stress (Nie et al., 2020). Analysis by Western Blot and immunofluorescence revealed that the expressions of NRF2, nucleus-NRF2 and HO-1 were further increased by rutaecarpine compared with the model group, while the expression of PGK1 was decreased by rutaecarpine (Figures 3A–G). We administered 20 mg/kg of CHX to mice along with 20 mg of rutaecarpine for the experiment and found that rutaecarpine was able to further inhibit PGK1 expression over time (Figures 3H,I).

### Rutaecarpine Improves the Viability of PC12 Cells Under Oxidative Stress

**FIGURE 4:**
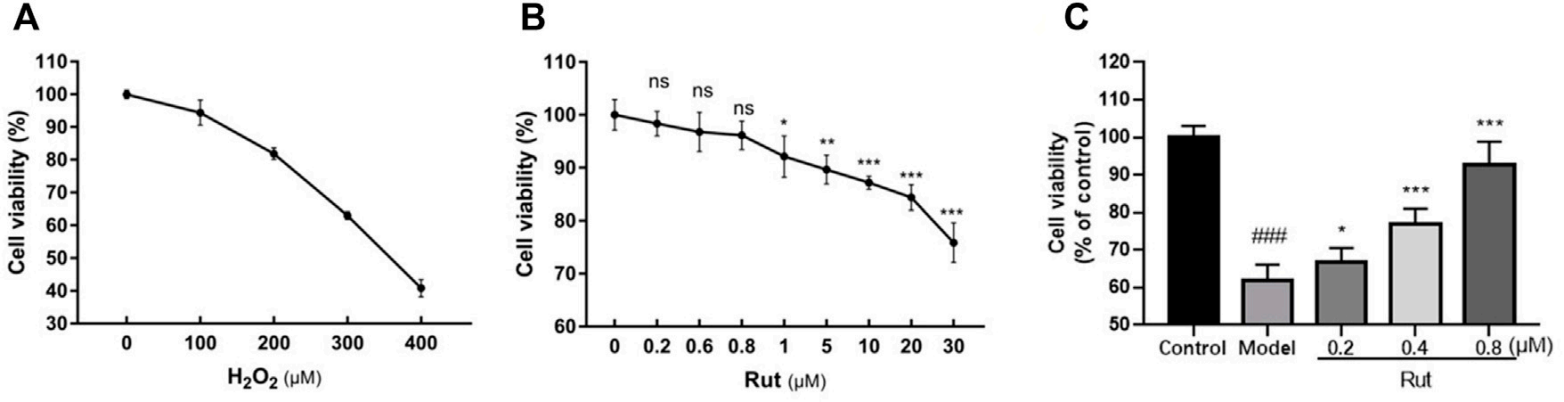
Rutaecarpine improves the viability of PC12 cells under oxidative stress. **(A)** Screening of H_2_O_2_ concentration for PC12 cells modeling. **(B)** Screening of rutaecarpine concentration for PC12 cells modeling. **(C)** The cell viability of PC12 cells in each group. *p < 0.05, **p < 0.01 and ***p < 0.001.

PC-12 cells are well known as an effective *in vitro* neuronal model and have been widely used to study the mechanism of neuronal damage in TBI (Bao et al., 2016). In Figures 4A,B, we confirmed the appropriate concentrations of H_2_O_2_ and rutaecarpine, at 300 μM for H_2_O_2_, 0.2, 0.4 and 0.8 μM for rutaecarpine, respectively. Next, the protective effect of rutaecaepine on cell viability was detected, and results suggested that rutaecaepine could effectively enhance the viability of PC12 cells under H_2_O_2_ stimulation (Figure 4C).

### Rutaecarpine Reduces H_2_O_2_-Stimulated Cellular Oxidative Stress Levels

**FIGURE 5:**
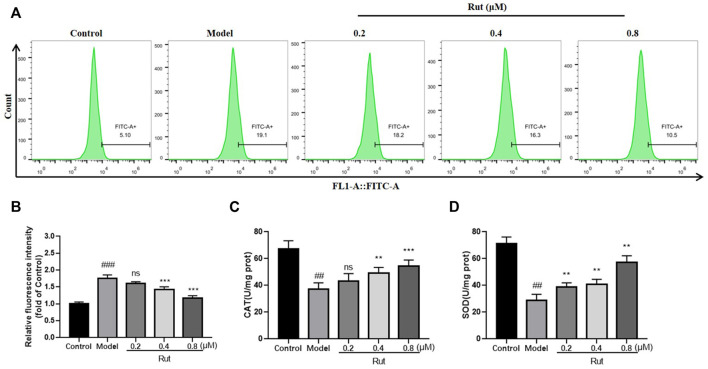
Rutaecarpine reduces oxidative stress levels in cells under H2O2-stimulated conditions. **(A)** The content of ROS in each group. **(B)** The relative fluorescence intensity of PC12 cells in each group. The activity of **(C)** CAT and **(D)** SOD of mice in each group. Compared with the control group, #*p* < 0.05 and ##*p* < 0.01; compared with the model group, *p < 0.05 and **p < 0.01.

The levels of ROS in PC12 cells subjected to oxidative stress in each group were showed in Figures 5A,B, and Figure 5B showed more visually that rutaecarpine at concentrations of 0.4 and 0.8 μM could significantly decrease the levels of ROS. The results of CAT and SOD of *in vitro* experiment were similar thgo that of *in vivo* experiment, with the oxidative stress-induced decrease in CAT and SOD significantly increased in the presence of rutaecarpine (Figures 5C,D).

### Rutaecarpine Reduces Apoptosis in PC12 Cells in Response to H_2_O_2_-Induced Oxidative Stress

**FIGURE 6:**
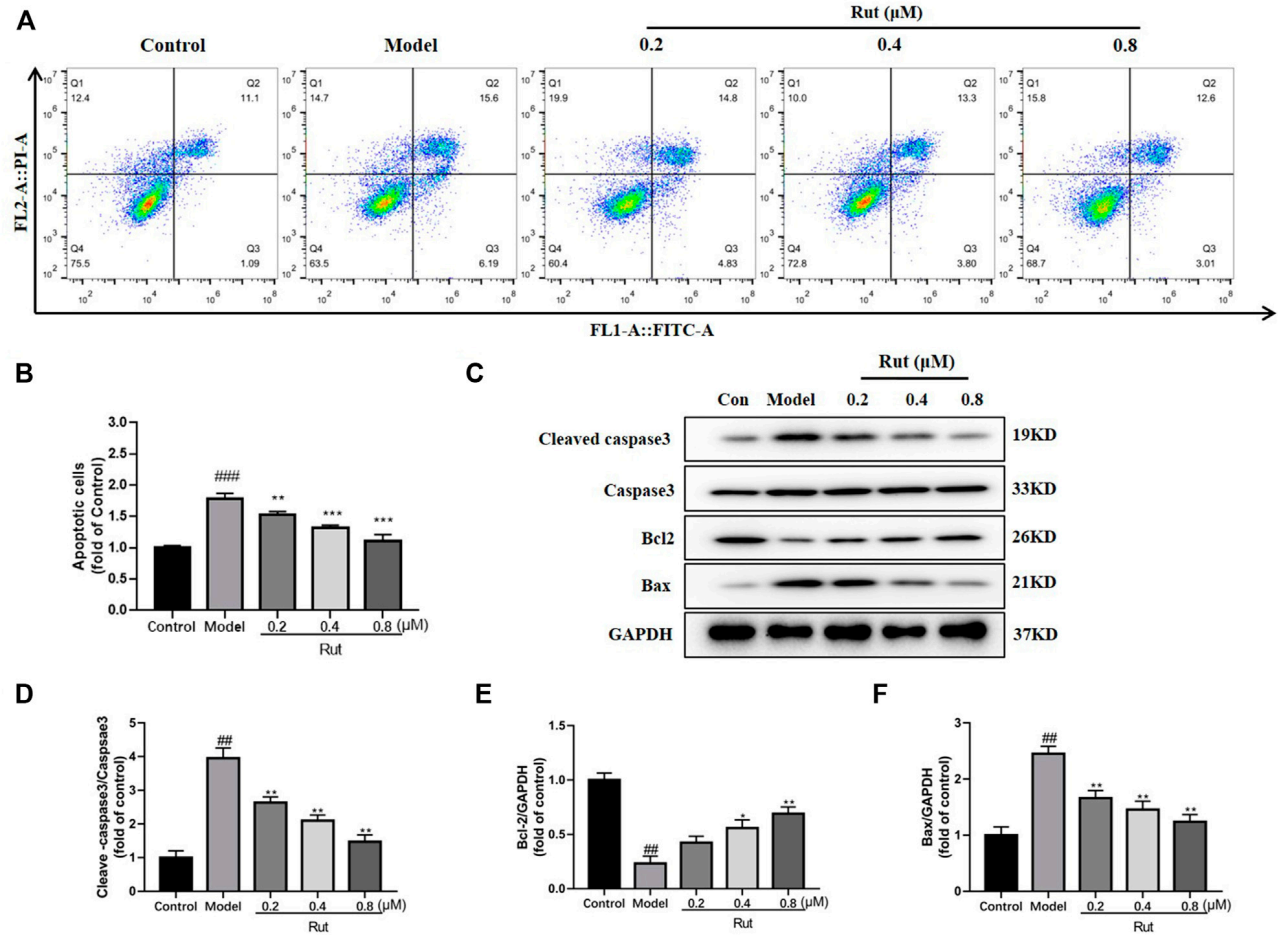
Rutaecarpine reduces apoptosis in PC12 cells in response to H2O2-induced oxidative stress. **(A)** Flow assay for apoptosis in each group. **(B)** The relative apoptosis cells in each group. **(C)** Expressions of apoptosis-related proteins cleaved-Caspase-3, Caspase-3, Bcl-2 and Bax in PC12 cells. Relative expressions of **(D)** cleaved-Caspase-3/Caspase-3, **(E)** Bcl-2 and **(F)** Bax of PC12 cells in each group. GAPDH was used as an internal control for proteins in **(C)**. Compared with the control group, #*p* < 0.05 and ##*p* < 0.01; compared with the model group, *p < 0.05 and **p < 0.01.

The proportion of apoptosis in PC12 cells under H_2_O_2_ stimulation treated or untreated with different concentrations of rutaecarpine were displayed in Figure 6A, and Figure 6B showed more visually that rutaecarpine could significantly decrease the apoptotic ratio of PC12 cells subjected to oxidative stress in a dose-dependent manner. In Figures 6C–F, the expressions of cleaved-caspase3/caspase3 and Bax were increased in the model group compared with the control group, while rutaecarpine abolished this trend; and the expression of Bcl-2 was decreased in the model group compared with the control group, while rutaecarpine reversed this change.

### Rutaecarpine Activates the NRF2 Pathway in PC12 Cells in Response to H_2_O_2_-Induced Oxidative Stress

**FIGURE 7:**
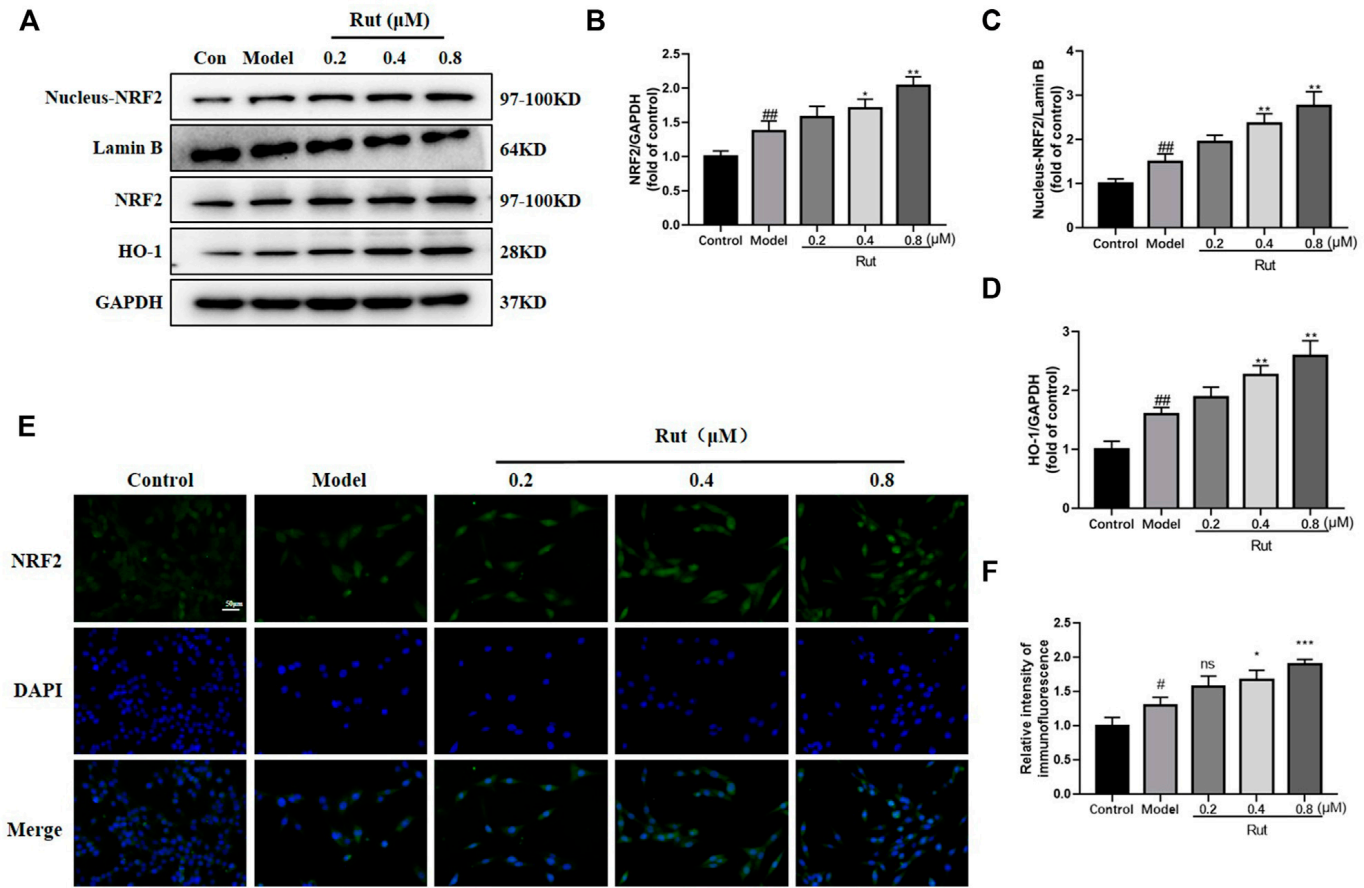
Rutaecarpine activates the NRF2 pathway in PC12 cells in response to H2O2-induced oxidative stress. **(A)** Expressions of NRF2 pathway-related proteins NRF2, Nucleus-NRF2 and HO-1 in PC12 cells. Relative expressions of **(B)** NRF2/GAPDH, **(C)** Nucleus-NRF2/Lamin B and **(D)** HO-1/GAPDH. **(E)** Represent images of NRF2 and DAPI immunofluorescence staining. **(F)** Relative intensity of immunofluorescence of NRF2. Lamin B was used as an internal control for Nucleus-NRF2, and GAPDH was used as an internal control for remaining proteins **(A)**. Compared with the control group, #*p* < 0.05 and ##*p* < 0.01; compared with the model group, *p < 0.05 and **p < 0.01.

As shown in Figures 7A–D, the expressions of NFR2, nucleus NFR2 and HO-1 were increased in the model group compared with the control group, and the expressions of NFR2, nucleus NFR2 and HO-1 were further increased in PC12 cells treated with rutaecarpine at concentrations of 0.4 and 0.8 μM. Moreover, the immunofluorescence intensity of NFR2 was significantly increased in rutaecarpine at concentrations of 0.4 and 0.8 μM compared with the models (Figures 7E,F).

### Rutaecarpine Regulates the Ubiquitinated Degradation of PGK1

**FIGURE 8:**
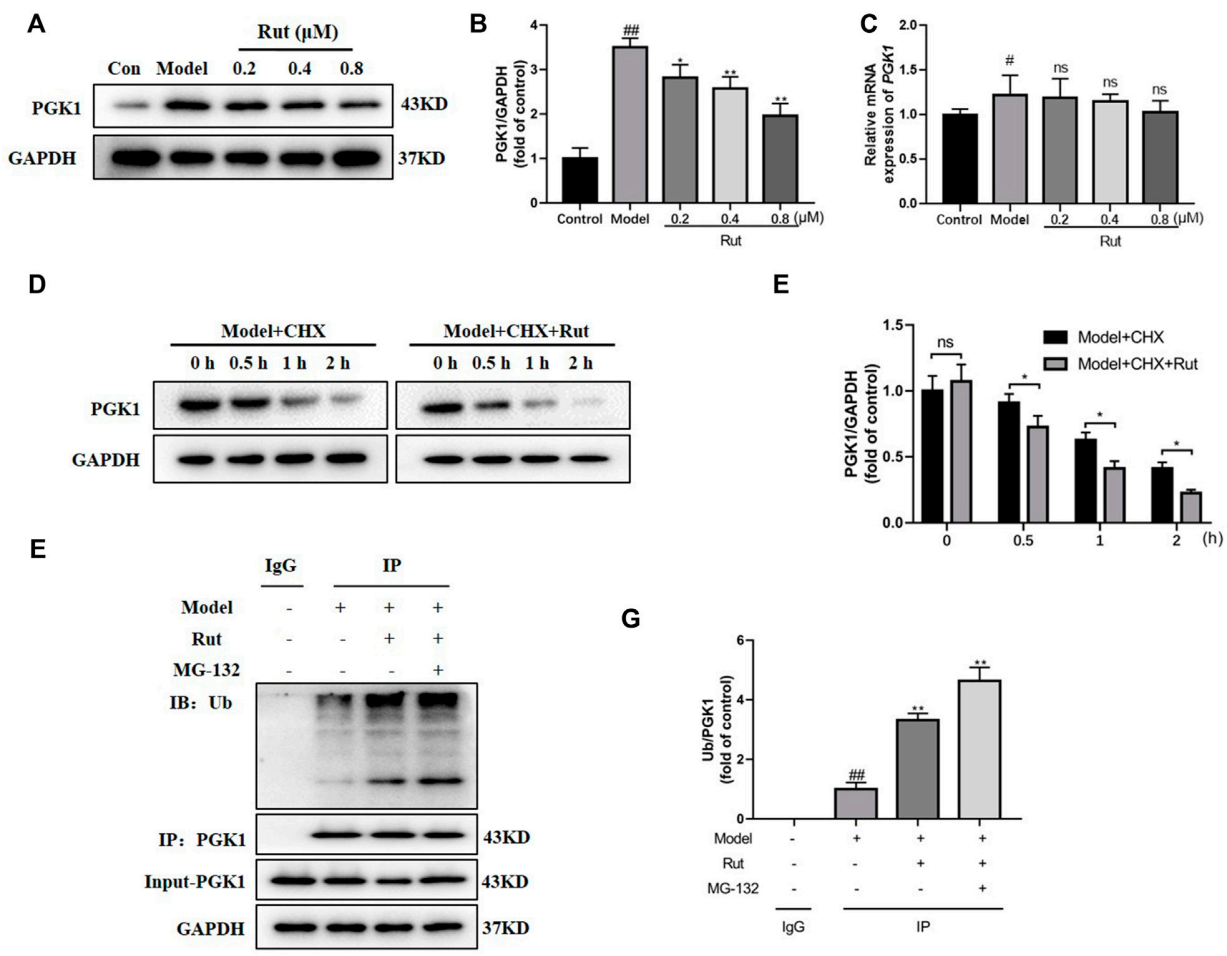
Rutaecarpine regulates the ubiquitinated degradation of PGK1. **(A)** Representative images of PGK1 in PC12 cells. **(B)** Relative expression of PGK1. **(C)** The relative mRNA expression of PGK1 in PC12 cells. **(D)** With increasing time, rutaecarpine further reduced the expression of PGK1. **(E)** The relative expression of PGK1 was presented in histograms. **(F)** Rutaecarpine further promotes PGK1 ubiquitination on the basis of MG-132. **(G)** The relative expression of PGK1 was presented in histograms. GAPDH was used as an internal control for proteins in **(A, D, F)**. Compared with the control group, #*p* < 0.05 and ##*p* < 0.01; compared with the model group, *p < 0.05 and **p < 0.01.

The protein expression of PGK1 was significantly decreased by rutaecarpine (*p* < 0.05, Figures 8A,B), while the difference in mRNA expression of PGK1 was not significant (Figure 8C). We added 50 μg/ml of CHX (protein synthesis inhibitor), along with 0.8 μM of rutaecarpine for the experiment and found that rutaecarpine was able to further inhibit PGK1 expression over time (Figures 8D,E). Under co-treatment with 25 μM MG-132 (proteasome inhibitor) and rutaecarpine, the inhibition of PGK1 by rutaecarpine was found to be achieved by promoting the ubiquitination of PGK1 (Figures 8F,G).

## Discussion

In addition to primary injury, TBI is accompanied by severe secondary injury, which is an important cause of neurological dysfunction after TBI. It is therefore crucial to investigate the molecular mechanisms of TBI and to find suitable drugs to inhibit this process. In the present study, we demonstrated that rutaecarpine treatment significantly ameliorated TBI-induced neuro-oxidative damage and preliminarily demonstrated that it may reduce apoptosis by reducing oxidative stress through the PGK1/NRF2 signaling pathway. Firstly, rutaecarpine treatment improved TBI-induced neurological dysfunction, reduced ROS levels and increased the antioxidant capacity of SOD and CAT, in addition to decreasing edema and apoptosis in mouse brain tissue. Secondly, rutaecarpine increased the survival of PC12 cell, reduced apoptosis and regulated the expressions of PGK1/NRF2/HO-1 pathway-related proteins. And furthermore, rutaecarpine was found to promote the ubiquitination of PGK1, thereby reducing the expression of PGK1.

Excessive oxidative stress can lead to neurological dysfunction, and the body’s internal antioxidant defence system maintains homeostasis that protects brain tissue from oxidative damage (Werner and Engelhard, 2007; Ansari et al., 2008). In TBI mice, the balance between oxidative stress and the antioxidant defence system was impaired and CAT and SOD levels were greatly reduced, whereas CAT and SOD levels were significantly increased with rutaecarpine administration. Similar results were seen in an *in vitro* cell model of TBI, where ROS levels were also reduced with increased CAT and SOD.

Oxidative stress also contributes to the activation of other secondary damage mechanisms, such as apoptosis (Abdul-Muneer et al., 2015). As a key pathological progression, apoptosis after TBI has attracted widespread attention, and inhibition of apoptosis in TBI models improves neurological dysfunction (Liu et al., 2018; Wang et al., 2019). In our mouse model of TBI, we examined the activation and expressions of apoptosis-related proteins in injured tissues and found increased expressions of cleaved-caspase 3 and Bax, and decreased expression of Bcl2. The expressions of NRF2 pathway-related proteins showed a significant increase in the expressions of NRF2/Lamin B, NRF2 and HO-1 in the TBI model group compared with the control group, which was further increased in the rutaecarpine treated groups and showed a dose-dependent manner of rutaecarpine. HO-1 catalyzes the catabolism of haemoglobin to ferrous iron, carbon monoxide and biliverdin (an important antioxidant enzyme) (Gao et al., 2021). On the one hand, the degradation of the haemoglobin moiety facilitates the prevention of its pro-oxidative effect; on the other hand, the byproduct biliverdin and its reduced bilirubin have an effective ROS scavenging activity (Zhu et al., 2008). The increase in the TBI model group may be a feedback increase following oxidative stress to the organism, while the reason for the increase in the rutaecaropine groups needs to be further explored.

In previous study, transfection of cells with PGK1-shRNA lentivirus reduced PGK1 expression, while NRF2 expression was increased and HO-1 expression of the NRF2 pathway-related gene was increased (Liang et al., 2019). This gives us a new idea, we examined the effect of rutaecarpine on PGK1. The protein expression of PGK1 decreased with increasing concentration of Rutaecarpine, but there was no change in its mRNA level, indicating that the effect of Rutaecarpine on PGK1 was proteolytic related. *In vitro* ubiquitination assay revealed that Rutaecarpine greatly promoted the ubiquitination of PGK1.

Although this is the first study of natural compound rutaecarpine to alleviate TBI via the PGK1/KEAP1/NFR2 pathway, it has several limitations. Firstly, this study found that rutaecarpine promoted PGK1 ubiquitination degradation to inhibit the PGK1/KEAP1/NRF2 pathway, thereby reducing oxidative stress levels to decrease apoptosis, but we only validated PGK1 ubiquitination and did not explore other possible underlying molecular mechanisms. Secondly, there was no exploration of whether ROS activates other cellular programs besides apoptosis. Furthermore, it should be validated with one more cell line in addition to the PC12 cell line.

## Conclusion

In conclusion, our study provides evidence that PGK1 plays an important role in the regulation of oxidative stress and apoptosis leading to TBI. Rutaecarpine can protect against neuronal oxidative damage by promoting the ubiquitination of PGK1, providing a new theoretical rationale for its clinical application.

## Data Availability

The raw data supporting the conclusion of this article will be made available by the authors, without undue reservation.

## References

[B1] Abdul-Muneer P. M. Chandra N. Haorah J. (2015). Interactions of Oxidative Stress and Neurovascular Inflammation in the Pathogenesis of Traumatic Brain Injury. Mol. Neurobiol. 51, 966–979. 10.1007/s12035-014-8752-3 24865512 PMC9420084

[B2] Ansari M. A. Roberts K. N. Scheff S. W. (2008). Oxidative Stress and Modification of Synaptic Proteins in hippocampus after Traumatic Brain Injury. Free Radic. Biol. Med. 45, 443–452. 10.1016/j.freeradbiomed.2008.04.038 18501200 PMC2586827

[B3] Bao H. Yang X. Zhuang Y. Huang Y. Wang T. Zhang M. (2016). The Effects of Poloxamer 188 on the Autophagy Induced by Traumatic Brain Injury. Neurosci. Lett. 634, 7–12. 10.1016/j.neulet.2016.09.052 27693566

[B4] Brown M. R. Sullivan P. G. Geddes J. W. (2006). Synaptic Mitochondria Are More Susceptible to Ca2+overload Than Nonsynaptic Mitochondria. J. Biol. Chem. 281, 11658–11668. 10.1074/jbc.M510303200 16517608

[B5] Chen J. Sanberg P. R. Li Y. Wang L. Lu M. Willing A. E. (2001). Intravenous Administration of Human Umbilical Cord Blood Reduces Behavioral Deficits after Stroke in Rats. Stroke 32, 2682–2688. 10.1161/hs1101.098367 11692034

[B6] Gao F. Wu X. Mao X. Niu F. Zhang B. Dong J. (2021). Astaxanthin Provides Neuroprotection in an Experimental Model of Traumatic Brain Injury via the Nrf2/HO-1 Pathway. Am. J. Transl Res. 13, 1483–1493. 33841672 PMC8014407

[B7] Han M. Hu L. Chen Y. (2019). Rutaecarpine May Improve Neuronal Injury, Inhibits Apoptosis, Inflammation and Oxidative Stress by Regulating the Expression of ERK1/2 and Nrf2/HO-1 Pathway in Rats with Cerebral Ischemia-Reperfusion Injury. Drug Des. Devel Ther. 13, 2923–2931. 10.2147/DDDT.S216156 PMC6708397 31692511

[B8] Hu Y. Yu C. Yao M. Wang L. Liang B. Zhang B. (2018). The PKCδ-Nrf2-ARE Signalling Pathway May Be Involved in Oxidative Stress in Arsenic-Induced Liver Damage in Rats. Environ. Toxicol. Pharmacol. 62, 79–87. 10.1016/j.etap.2018.05.012 29986281

[B9] Launey Y. Fryer T. D. Hong Y. T. Steiner L. A. Nortje J. Veenith T. V. (2020). Spatial and Temporal Pattern of Ischemia and Abnormal Vascular Function Following Traumatic Brain Injury. JAMA Neurol. 77, 339–349. 10.1001/jamaneurol.2019.3854 31710336 PMC6865302

[B10] Liang J. Zhang X. Y. Zhen Y. F. Chen C. Tan H. Hu J. (2019). PGK1 Depletion Activates Nrf2 Signaling to Protect Human Osteoblasts from Dexamethasone. Cell Death Dis 10, 888. 10.1038/s41419-019-2112-1 31767834 PMC6877585

[B11] Liu Y. Bao Z. Xu X. Chao H. Lin C. Li Z. (2017). Extracellular Signal-Regulated Kinase/Nuclear Factor-Erythroid2-like2/Heme Oxygenase-1 Pathway-Mediated Mitophagy Alleviates Traumatic Brain Injury-Induced Intestinal Mucosa Damage and Epithelial Barrier Dysfunction. J. Neurotrauma. 34, 2119–2131. 10.1089/neu.2016.4764 28093052

[B12] Liu Z. M. Chen Q. X. Chen Z. B. Tian D. F. Li M. C. Wang J. M. (2018). RIP3 Deficiency Protects against Traumatic Brain Injury (TBI) through Suppressing Oxidative Stress, Inflammation and Apoptosis: Dependent on AMPK Pathway. Biochem. Biophys. Res. Commun. 499, 112–119. 10.1016/j.bbrc.2018.02.150 29470982

[B13] Marmarou A. (2003). Pathophysiology of Traumatic Brain Edema: Current Concepts. Acta Neurochir. Suppl. 86, 7–10. 10.1007/978-3-7091-0651-8_2 14753394

[B14] McGinn M. J. Povlishock J. T. (2016). Pathophysiology of Traumatic Brain Injury. Neurosurg. Clin. N. Am. 27, 397–407. 10.1016/j.nec.2016.06.002 27637392

[B15] Mustafa A. G. Alshboul O. A. (2013). Pathophysiology of Traumatic Brain Injury. Neurosciences (Riyadh) 18, 222–234. 23887212

[B16] Namani A. Li Y. Wang X. J. Tang X. (2014). Modulation of NRF2 Signaling Pathway by Nuclear Receptors: Implications for Cancer. Biochim. Biophys. Acta 1843, 1875–1885. 10.1016/j.bbamcr.2014.05.003 24851839

[B17] Namani A. Matiur Rahaman M. Chen M. Tang X. (2018). Gene-expression Signature Regulated by the KEAP1-NRF2-CUL3 axis Is Associated with a Poor Prognosis in Head and Neck Squamous Cell Cancer. BMC Cancer 18, 46. 10.1186/s12885-017-3907-z 29306329 PMC5756380

[B18] Nie H. Ju H. Fan J. Shi X. Cheng Y. Cang X. (2020). O-GlcNAcylation of PGK1 Coordinates Glycolysis and TCA Cycle to Promote Tumor Growth. Nat. Commun. 11, 36. 10.1038/s41467-019-13601-8 31911580 PMC6946671

[B19] Pearn M. L. Niesman I. R. Egawa J. Sawada A. Almenar-Queralt A. Shah S. B. (2017). Pathophysiology Associated with Traumatic Brain Injury: Current Treatments and Potential Novel Therapeutics. Cell Mol. Neurobiol. 37, 571–585. 10.1007/s10571-016-0400-1 27383839 PMC11482200

[B20] Shah S. Z. A. Zhao D. Hussain T. Sabir N. Mangi M. H. Yang L. (2018). p62-Keap1-NRF2-ARE Pathway: A Contentious Player for Selective Targeting of Autophagy, Oxidative Stress and Mitochondrial Dysfunction in Prion Diseases. Front. Mol. Neurosci. 11, 310. 10.3389/fnmol.2018.00310 30337853 PMC6180192

[B21] Skolnick B. E. Maas A. I. Narayan R. K. van der Hoop R. G. MacAllister T. Ward J. D. (2014). A Clinical Trial of Progesterone for Severe Traumatic Brain Injury. N. Engl. J. Med. 371, 2467–2476. 10.1056/NEJMoa1411090 25493978

[B22] Wang W. T. Sun L. Sun C. H. (2019). PDIA3-regulted Inflammation and Oxidative Stress Contribute to the Traumatic Brain Injury (TBI) in Mice. Biochem. Biophys. Res. Commun. 518, 657–663. 10.1016/j.bbrc.2019.08.100 31466719

[B23] Werner C. Engelhard K. (2007). Pathophysiology of Traumatic Brain Injury. Br. J. Anaesth. 99, 4–9. 10.1093/bja/aem131 17573392

[B24] Wu L. Zhang K. Hu G. Yan H. Xie C. Wu X. (2014). Inflammatory Response and Neuronal Necrosis in Rats with Cerebral Ischemia. Neural Regen. Res. 9, 1753–1762. 10.4103/1673-5374.143419 25422636 PMC4238163

[B25] Yan C. Zhang J. Wang S. Xue G. Hou Y. (2013). Neuroprotective Effects of Rutaecarpine on Cerebral Ischemia Reperfusion Injury. Neural Regen. Res. 8, 2030–2038. 10.3969/j.issn.1673-5374.2013.22.002 25206511 PMC4146067

[B26] Zhang Y. Zhang Z. G. Chopp M. Meng Y. Zhang L. Mahmood A. (2017). Treatment of Traumatic Brain Injury in Rats with N-Acetyl-Seryl-Aspartyl-Lysyl-Proline. J. Neurosurg. 126, 782–795. 10.3171/2016.3.JNS152699 28245754 PMC5116431

[B27] Zhang Y. N. Yang Y. F. Yang X. W. (2018). Blood-brain Barrier Permeability and Neuroprotective Effects of Three Main Alkaloids from the Fruits of Euodia Rutaecarpa with MDCK-pHaMDR Cell Monolayer and PC12 Cell Line. Biomed. Pharmacother. 98, 82–87. 10.1016/j.biopha.2017.12.017 29245070

[B28] Zhu H. Jia Z. Misra B. R. Zhang L. Cao Z. Yamamoto M. (2008). Nuclear Factor E2-Related Factor 2-dependent Myocardiac Cytoprotection against Oxidative and Electrophilic Stress. Cardiovasc. Toxicol. 8, 71–85. 10.1007/s12012-008-9016-0 18463988

